# A protocol to evaluate the impact of involvement of older people with dementia and age-related hearing and/or vision impairment in a multi-site European research study

**DOI:** 10.1186/s40900-018-0128-9

**Published:** 2018-11-22

**Authors:** Jahanara Miah, Piers Dawes, Iracema Leroi, Suzanne Parsons, Bella Starling

**Affiliations:** 1 Division of Neuroscience and Experimental Psychology, University of Manchester, Jean McFarlane Building, Oxford Road, Manchester, M13 9PL UK; 2 Manchester Centre for Audiology and Deafness (ManCAD), Manchester Academic Health Science Centre, University of Manchester, Oxford Road, Manchester, M13 9PL UK; 3 Public Programmes Team, Research and Innovation Division, Manchester University NHS Foundation Trust and The University of Manchester, 29 Grafton Street, Manchester, M13 9WU UK

**Keywords:** Patient and public involvement, Older people, Dementia, Hearing, Vision, Europe, Research user groups, Protocol

## Abstract

**Plain English summary:**

Involving older people with dementia in research is increasingly recognised as important to ensure that research is relevant and beneficial for older people with dementia. But researchers need to know how best to involve older people with dementia and to be able to show the benefits of involving older people with dementia in dementia research.

This paper describes a research plan to explore the involvement of older people with dementia and age-related hearing and/or vision impairment in a European research project investigating the combined impact of dementia with hearing and/or vision impairment. We set up four Research User Groups (RUGs) of older people with dementia with age-related hearing and/or vision impairment and their carers based in the UK, France, Cyprus and Greece to advise our researchers. We provided training to group members to support their input to the research.

We will use a questionnaire and interview people in our RUGs to understand what they thought of the training and their experiences of being part of the RUG. We will also interview researchers to understand if they thought the advice from the groups was useful.

This study will help us to understand how to effectively involve older people with dementia and age-related hearing and/or vision impairment in research and what the benefits of involving older people with dementia in research are.

**Abstract:**

**Background**

Research to prevent and treat dementia is an international priority. Involvement of older people with dementia in the research is important to ensure the relevance and utility of the research outcomes in clinical practice to them. Efforts to involve such people in research are growing due to increased recognition of the usefulness of incorporating the views of older people with dementia into the research process. Research User Groups (RUGs) of older people with dementia and carers for people with dementia were set up in UK (Manchester), France (Nice), Cyprus (Nicosia) and Greece (Athens) to advise on the research. We report a protocol for a study which aims to evaluate i) the perceptions of RUG members of the usefulness of Research Awareness Training that was provided to support their involvement in the research and ii) perceived impacts of the involvement of older people with dementia and age-related hearing and/or vision impairment on research from the point of view of RUG members and researchers.

**Methods**

Both qualitative and quantitative methods will be used to evaluate the acceptability, appropriateness and satisfaction with Research Awareness Training and the perceived impact of involvement of RUGs on research. Focus groups interviews with RUG members and one to one interviews with both RUG members (*n* = 24) and researchers (*n* = 6) will be conducted to understand the perceived impacts of patient and public involvement on research from the point of view of older people with dementia, carers and researchers. Any comparative differences in cultural, attitudinal and environmental differences between RUGs in outcomes of training and impact across the four European sites will be reported.

**Discussion**

This study is unique in its exploration of the impact of the involvement of older people with dementia and age-related hearing and/or vision impairment in a large multi-site European dementia research study. This work will be crucial in informing understanding of how to effectively involve older people with dementia and age-related hearing and/or vision impairment and carers in dementia research to ensure research addresses the needs and priorities of older people with dementia and age-related hearing and/or vision impairment.

**Electronic supplementary material:**

The online version of this article (10.1186/s40900-018-0128-9) contains supplementary material, which is available to authorized users.

## Background

Research to prevent and treat dementia as well as improve quality of life for people with dementia is an international priority [1]. Involvement of people with dementia in research is important to ensure the relevance and utility of the research outcomes in clinical practice [2, 3]. There is increasing recognition of the need to involve people with dementia in research due to an increased focus on person-centred care and the attendant requirement to take the views of people with dementia into account [4–6]. There is also evidence to suggest the involvement of people with dementia in research has an impact on research, researchers, the organisation and the person involved in the research [7]. Consequently, there is growing interest in understanding how to involve people with dementia as active partners in research [4–6].

The rationale for patient and public involvement in research in general is that people with lived experience of a health condition or treatment can offer valuable perspectives to the research process [8, 9]. INVOLVE, the coordinating centre for patient and public involvement in the UK’s National Health Service defines involvement as “research being carried out ‘with’ or ‘by’ members of the public rather than ‘to’, ‘about’, or ‘for them”’ [8]. Involvement can take a number of forms including consultation (e.g. being involved in prioritising research), collaboration (e.g. acting as an advisor on a research project) or user control (e.g. acting as a service user researcher and carrying out the research) [8]. People with dementia may be involved in all stages of the research process [6, 8, 9]. For example, people with dementia may be involved in identifying research priorities relevant to patients and carers [10, 11], developing funding applications [12], enhancing the relevance and feasibility of research to develop effective interventions [13], identifying potential barriers at an early stage in the research [14, 15], contributing to the interpretation of findings [16, 17], and the dissemination of research results [18, 19]. Being involved in research may also provide benefits to people with dementia and their carers including increasing knowledge, enhancing skills, developing networks and new opportunities for involvement [20], meeting with and hearing the opinions of others with dementia [19, 21], improving self-confidence, and offering opportunities to contribute to dementia research in a forum where the views of people with dementia are valued [21].

### Study context

**Fig. 1:**
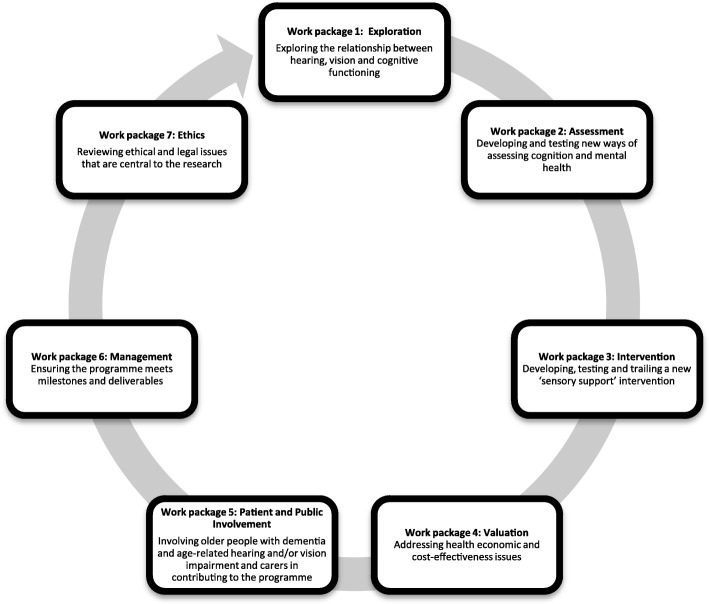
SENSE-Cog work packages. SENSE-Cog aims to investigate the combined impact of dementia and age-related hearing and/or vision impairment and to develop new tools to improve quality of life for older Europeans. The project started in January 2016 and will run until December 2020. Involvement of older people with dementia in SENSE-Cog is a significant part of the overall work programme

This study is being conducted as part of a larger project, ‘SENSE-Cog’, which aims to investigate the combined impact of dementia and age-related hearing and/or vision impairment and to develop new tools to improve quality of life for older Europeans [22]. SENSE-Cog is funded by the European Research Council’s Horizon 2020 research and innovation programme. The project started in January 2016 and will run until December 2020. Involvement of older people with dementia in SENSE-Cog is a significant part of the overall work programme (Fig. 1).

This paper focuses on SENSE-Cog work package five, ‘patient and public involvement’. Older people with dementia and age-related hearing and/or vision impairment and carers are involved in SENSE-Cog research via Research User Groups (RUGs). RUG membership was constrained to people over the age of 65 years to reflect the target population of the SENSE-Cog research study. RUGs are groups of people who are brought together and supported to give their views and input into SENSE-Cog research. Four RUGs have been established at SENSE-Cog partner sites in Manchester, Nice, Nicosia and Athens. RUGs consist of 7–10 older people with dementia and age-related hearing and/or vision impairment and/or their carers.

Prior to recruiting RUG members, we consulted with a group of older people with dementia and their carers affiliated with Manchester Institute for Collaborative Research on Ageing (MICRA) in a focus group discussion to comment on a draft recruitment flyer for RUG members, we asked participants to comment on the wording, presentation and readability of the flyer. We also asked them to advise us on where to advertise the flyers, and on considerations when setting up meetings for older people with dementia and age-related hearing and/or vision impairment. The draft flyer was revised following the focus group recommendations and translated into French and Greek for the RUG sites in Nice, Nicosia and Athens. The flyers were advertised locally in each RUG site through local organisations and networks working with older people with dementia and older people with dementia and age-related hearing and/or vision impairment. For example, for the Manchester RUG recruitment, flyers were distributed via an email list members of the general public interested in aging research, local memory clinics, and charity organisations including the Alzheimer’s Society, Age UK, Manchester Dementia Action Alliance and Action on Hearing Loss. We received relatively few queries about involvement in general and no queries from anyone from Black, Asian and minority ethnic or underserved communities. On reflection, this recruitment method was not very effective in reaching a diverse range of potential participants. A more targeted community outreach approach such as in-person talks to local dementia support groups, organisations working with Black, Asian and minority ethnic or underserved communities could have been a better method of recruiting a more diverse RUG.

For all RUGs, introductory meetings were held to allow members to decide on how the group should operate and function, e.g. the frequency, length and format of meetings and training delivery. All RUGs are supported and managed by a local patient and public involvement coordinator. Local patient and public involvement coordinators were identified among the research team in each study site. Patient and public involvement coordinators have a background in research with people with dementia, and have experience of working with older adults in research settings. All patient and public involvement coordinators speak English, and communicate in English with the patient and public involvement work package coordinator in Manchester, UK. The role of the patient and public involvement coordinator is to arrange meetings, liaise with researchers and develop activities to support the groups’ involvement in the research.

RUGs meet face-to-face every 3 months, as well as ad-hoc individual meetings with up to 2 RUG members (with the option of postal, telephone, e-mail communication, or in person) for any additional involvement tasks identified by the research team that require patient and public involvement. A key element of the patient and public involvement coordinator’s work is the on-going monitoring of the support requirements of each RUG member. During the introductory meetings the patient and public involvement coordinators completed a support and learning needs form [see Additional file 1] with individual RUG members to understand the different support and learning needs within the group. Support and learning needs are continually revised by the patient and public involvement coordinators by telephone conversations 2 weeks before the RUG meeting. Telephone contact was also preferred by those with hearing problems as RUG members with hearing problems had amplified telephones at home. Regular contact with the RUG members helps the patient and public involvement coordinator to be aware of RUG members’ changing needs so that appropriate support can be provided to facilitate involvement. Patient and public involvement coordinators have links with local support services (such as Admiral Nurses and the Alzheimer’s Society in the UK) that can provide RUG members with additional support if needed. Patient and public involvement coordinators used information from the support and learning needs forms to put in place individual support arrangements to facilitate each person’s involvement. For example, for those with vision problems, patient and public involvement coordinators position themselves close to the person and keep still while talking. People with vision problems are provided with training and RUG materials in large font black print on yellow paper. Patient and public involvement coordinators also use verbal cues to direct people’s attention to specific written materials, for example “Look at the first paragraph on page 2 of the hand-out” rather than saying “Look at the hand-out”.

### Aims and objectives of the study

This study aims to evaluate i) the acceptability and perceived outcomes of Research Awareness Training that was provided to support the involvement of older people with dementia and age-related hearing and/or vision impairment in the research and ii) the perceived impacts of the involvement of older people with dementia and age-related hearing and/or vision impairment on the research from the point of view of RUG members and researchers (Study time frame: Table 1).

**Table 1:** Study time frame for Manchester, Nicosia, Nice and Athens sites

	Jan 2017	April 2017	Jul 2017	Oct 2017	Jan 2018	April 2018	Oct 2018	Jan 2019
SENSE-Cog project timeline (project month)	13	16	19	22	25	28	34	37
Delivery of Research Awareness Training								
TARS questionnaires								
Semi structured interviews with RUG members and researchers								
Focus group interviews								

### Evaluating acceptability and perceived outcomes of research awareness training

#### Sample

All RUG members involved in the Research Awareness Training in Manchester, Nicosia, Nice and Athens will be invited to take part in the evaluation of the training. The evaluation will be completed by up to 10 (people) RUG members in each study site (Manchester, Nicosia, Nice and Athens).

#### Research awareness training

**Fig. 2:**
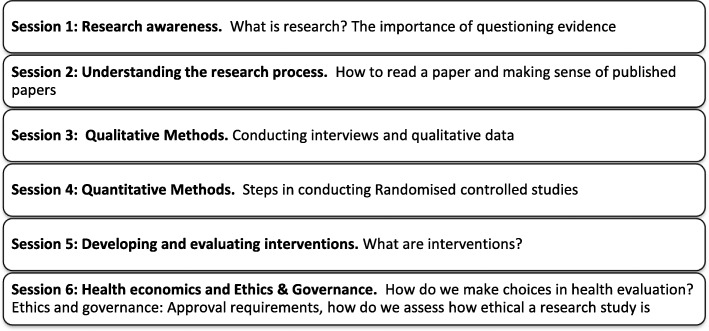
Adapted Research Awareness Training. The adapted Research Awareness Training for RUG members consists of 6 one hour sessions focussing on key research concepts that are relevant for SENSE-Cog. The training was developed following discussion with RUG members in each study site. RUG members’ preference was for shorter, bite-sized training delivered as needed

The Research Awareness Training (Fig. 2) was initially developed as part of the Enhancing the Quality of User Involved Care Planning (EQUIP) [23] programme to give UK National Health Service mental health service users and caregivers an understanding of research and research terminology to support them in working as co-researchers on a mental health research project. EQUIP training involved a 6-day research methods course. However we adapted the training to 6 one hour sessions focussing on key research concepts that were relevant for SENSE-Cog. A shorter version of Research Awareness Training than the original 6 day program was developed following discussion with RUG members in each study site during the introductory meetings. RUG members’ preference was for shorter, bite-sized training delivered as needed. Shorter, bite-sized training also facilitated participation of people with memory difficulties.

Patient and public involvement coordinators for each RUG completed a three day training course in English (Table 2) to orient them to the content of the Research Awareness Training and describe how training should be delivered to RUGs (Table 2). Discussion of the principles of patient and public involvement training and orientation to the SENSE-Cog project was delivered by the Public Programmes Team in Manchester [24]. The Public Programmes Team is a specialist unit advising on and delivering patient and public involvement and engagement in national and international health research [24]. Research Awareness Training for patient and public involvement coordinators was delivered by Professor Karina Lovell and colleagues at the University of Manchester based on a ‘train the trainer model’ [23] to enable the patient and public involvement coordinators to adapt the Research Awareness Training for local implementation appropriate for RUG member’s language, hearing and/or vision impairments.

**Table 2:** Patient and public involvement coordinators training

Day 1 Principles of patient and public involvement	Day 2 Research Awareness Training	Day 3 Research Awareness Training
Overview of SENSE-Cog and work package 5 objectives to enable patient and public involvement coordinators to establish and support local RUG members and orientate the members to the SENSE-Cog programme and work package 5 objectives.	Good facilitation skills and techniques. Managing difficult situations; case scenarios. Research Awareness Training topics.	Research Awareness Training topics continued. Adaptation plans for Research Awareness Training topics, and cultural differences to take into consideration.

Patient and public involvement coordinators deliver Research Awareness Training to RUGs using a two-way flow interactive discussion. Two-way flow interactive discussion involves an exchange of ideas where both facilitator and RUG members are active and can prompt one another in the discussion of a particular research topic. Each Research Awareness Training session lasts around 60 min. Each training session is delivered to support and complement RUG members’ involvement in specific aspects of SENSE-Cog research on a needs basis. For example, Research Awareness Training on qualitative methods is offered immediately prior to the RUGs reviewing a question related to qualitative aspects of SENSE-Cog research.

#### Measures

Quantitative and qualitative methods will be used to evaluate the experience of Research Awareness Training for RUG members.

Training Acceptability Rating Scale (TARS) [25].

The TARS [25] was selected for evaluating the training, as the TARS has previously been used to evaluate Research Awareness Training for patient and public involvement volunteers [26, 27]. The TARS is a self-administered paper questionnaire and takes approximately 5–10 min to complete. The TARS consists of three parts: the first concentrating on appropriateness and acceptability of the training, the second covering effectiveness and competency of the trainers. The first two parts contain items rated on a four-point Likert scale, ranging from ‘Not at all’ (score 1) to ‘A great deal’ (score 4). The third and final part includes three open ended questions for RUG members to identify the most useful elements of the training, suggest changes and add additional comments. The Research Awareness Training materials and TARS was translated into Greek and French using the ‘forward-translations and back-translations’ procedure [28].

Semi structured interview and focus group interview.

RUG members who have completed the Research Awareness Training will be approached to take part in a semi-structured interview [see Additional file 2] or focus group interview [see Additional file 3] to give their impressions of the training overall, indicate what insights, understanding and skills they had acquired and how they have applied the knowledge and skills provided by the training within the SENSE-Cog programme.

#### Procedure

Training sessions will be carried out approximately every 3 months. There is some flexibility in the scheduling of training delivery according to local requirements. For example, one training session in Cyprus was postponed from July to September due to very hot summer weather. Participants will complete the TARS questionnaire at the end of each training session to ensure that those with cognitive or memory impairment can provide immediate feedback for each training session. The semi-structured interview will be conducted once participants have completed all 6 training sessions in order to obtain each RUG members’ overall impressions of training.

### Perceived impacts of the involvement of older people with dementia on the research from the point of view of RUG members and researchers

#### Sample

Twenty-four one to one interviews with RUG members (6 at each site) and 6 one to one interviews with SENSE-Cog researchers will be conducted across all sites. The final sample size will be determined by data saturation [29–31], although we anticipate that a sample of approximately 6 interviews will be required.

Additional focus group interviews to capture RUG members’ views on the on-going RAT and their experience as a RUG member and their perceived impact on the research process, will allow us to reflect on our programme’s progress towards achieving its objectives and make any changes on an on-going basis, such as change of venue or working approach with the RUG members. We will aim to maximise diversity in the study sample to increase the validity and transferability of research findings to other settings. The sample of RUG members will include (i) men and women ages 65 years and above (ii) older people with dementia (iii) older people with dementia and age-related hearing and/or vision impairment (iv) carers with age related hearing and/ or vision impairment. RUG membership was constrained to include only people over the age of 65 due to the focus on older adults in the SENSE-Cog project. Younger people with dementia and younger carers will not therefore be included in the research evaluation. The sample of researchers will include i) researchers at different levels (i.e. work package leads and research assistants), ii) researchers in different SENSE-Cog work packages (see Fig. 1) who have worked with the RUGs and iii) different countries.

#### Measures

One to one semi structured interviews and focus group interviews will be conducted with RUG members by the patient and public involvement coordinators in each site to capture RUG members’ impression of the training and of their experience as a RUG member and their perceived impact on the research process. One to one semi structured interviews with SENSE-Cog researchers [see Additional file 4] across all sites will be conducted by the project coordinator based in Manchester to assess SENSE-Cog researchers experience of working with RUGs and the impacts that RUGs have had on the research process.

#### Procedure

Eight focus group interviews will be conducted in total with RUG members (2 focus groups in each RUG site). Focus group interviews will take place at approximately 22 months and 37 months following the commencement of RUG activities. These time points were selected in order to record RUG members’ impressions at the approximate mid-point and near the end of involvement in the research to capture people’s views and opinions during on-going involvement in research. In addition, we chose to collect focus group data at more than one point due to the age of RUG members and the progressive nature of dementia. RUG participants may drop in or out of involvement if their condition fluctuates or if RUG members can no longer provide on-going informed consent to participate in the research evaluation of their involvement. One to one interviews with RUG members will be conducted at approximately 34 months. Similarly.

One to one interviews with SENSE-Cog researchers will be conducted at approximately 34 months in order to capture researcher’s perceptions of RUG involvement near the end of the research program.

### Data analysis

TARS [25] data will be analysed at group level using SPSS (IBM, Armonk, NY, United States) to generate descriptive statistics. The open ended questions from the TARS will be analysed qualitatively, as for the interview data below.

One to one interviews and focus group interviews will be audio-recorded. Audio-recordings in Nice, Nicosia and Athens will be transcribed verbatim in the local languages and then translated by each local patient and public involvement coordinator. The data from all 4 sites will be analysed by the lead Study Coordinator in Manchester using NVIVO qualitative software (QSR International Pty Ltd., Victoria, Australia). The FRAMEWORK method [32] will be used for data analysis. The FRAMEWORK method allows in-depth analysis of key themes across the whole datasets, as well as between individual accounts; as it uses existing topic guide as a starting point for the framework, whereas other thematic approaches may just generate themes directly from the data. In addition, the framework method allows the summaries of data across each case and theme or subtheme into charts using Excel which makes the data accessible to a wider research team.

Data from interviews from each site will firstly be analysed separately to produce themes that are relevant to each site. Coding that is conceptually and characteristically noteworthy will be highlighted, rather than focusing just on frequency of occurrence [32, 33]. After separate analysis of interview data for each site, themes identified in separate analyses for each site will then be evaluated for common themes across all sites, as well as identification of distinct issues between sites. Analysis across sites will involve a systematic approach for the patient and public involvement coordinators in each sites to refine the themes, classify them into categories and draw conclusions about differences between sites.

The format of the Guidance for Reporting Involvement of Patients and the Public (GRIPP2) [34] reporting checklists will be used to report the implementation and evaluation of patient and public involvement in this study and we will share our findings on the evaluation of the Research Awareness Training via peer reviewed publication once it is complete.

### Ethics

The study received ethical approval from Manchester University Research Ethics Committee. Additional ethical approvals were sought and obtained for each study site (Nicosia, Nice, Athens), as relevant to local arrangements.

A key ethical issue when working with people with dementia is informed consent, especially how researchers establish whether people have capacity for informed consent and how any changes in capacity are identified. According to the Mental Capacity Act (2005) [35], people should be assumed to have capacity unless otherwise demonstrated. The capacity of participants with cognitive impairment to consent to participate (provided that they have clear and appropriate study information and enough time to make decisions) will be assessed on an on-going basis. All staff members involved in this research completed training in assessing capacity in research.

To support the informed participation in the research, RUG members provided input into the development of easy access user friendly versions of the participant information sheet and consent form [see Additional file 5]. In cases where RUG members are unable to complete the consent form due to low vision, the consent form is read out verbatim. RUG member may then agree verbally to participate, and the patient and public involvement coordinator signs the consent form on behalf of the RUG member, which is then witnessed and countersigned by another SENSE-Cog project researcher.

The patient and public involvement coordinators at each site contact the RUG members via telephone 2 weeks before each training session to establish their capacity to consent, and their understanding of their involvement in the training and the evaluation study. The patient and public involvement coordinator assesses whether RUG members can retain the information discussed with them over telephone or in person at the time of discussion by asking the RUG member to repeat the information explained to them. Pre-meetings take place before each training session or RUG meeting to give the patient and public involvement coordinator the opportunity to speak with the participant again to establish their capacity for consent and involvement. A similar approach will be taken prior to the one to one interviews and focus group interviews with RUG members.

## Discussion

Dementia research is an international priority. To ensure the relevance of research outcomes to older people with dementia, it is vital the views of older people with dementia are taken into account when designing research [2, 3, 6–8]. There is a small but growing literature on the methodology and impacts of involving people with dementia in dementia research [7, 36]. Previous reviews of PPI in health research have recognised a lack of reporting of the impact and benefits of PPI, and where studies have described the impact of PPI, the quality of the evidence is low [37–40]. The lack of good quality research on the impacts and benefits of PPI necessitates the need for evidence-based models of good practice of patient and public involvement in health research in general as well as evidence for the impacts and benefits of involvement [37–41]. The need is particularly acute in the case of involving older people with dementia and age-related hearing and/or vision impairment in health research. Very few studies have described and evaluated patient and public involvement with older people with dementia [7, 19, 21, 36, 42–44].

In the present study, we made particular methodological choices to facilitate the participation of older people with dementia and age-related hearing and/or vision impairment in the research evaluation of Research Awareness Training and the impact of RUGs on the SENSE-Cog research project. We chose a combination of questionnaires, focus groups and one to one interviews, because people with dementia have previously participated in research to investigate their opinions and feelings through the use of questionnaires, focus groups and one to one interviews [44–48]. We also employed procedures that maximise participation of people with dementia in research [6, 49–52]. For example, questionnaires were completed by RUG members at the end of each training session, minimising the demand on memory to recall past experiences with training. Completing the questionnaires after each training session also allowed participants to ask for clarification from the patient and public involvement coordinators if they required it. We took a flexible approach for the first sets of focus group interviews, based on whether or not the participant wanted to contribute in the group discussion on the day, with quiet rooms available for rest breaks.

Similarly one to one interviews enable the interviewer to explore opinions in-depth, pick up on non-verbal interactions, check the comprehension of questions and allow participants to take time to reflect and respond [48, 49, 51]. We are aware that people with dementia may not be able to participate in interviews of long duration [53]. We therefore have planned to take a flexible approach and conduct interviews based on whether or not the RUG participant wants to complete the interview on the day. We will attempt to minimise the overall time of the interview and/or plan in regular breaks during the interviews with quiet rooms available to offer opportunities for rest breaks. The patient and public involvement coordinators will conduct the one to one interviews, as they have an established relationship with RUG members and are aware of the needs and style of communication for each member. Patient and public involvement coordinators will plan in extra time before and after the interview to help make the participant feel comfortable [6, 49].

This protocol sets out to evaluate training to support the involvement of older people with dementia and age-related hearing and/or vision impairment and the impact of their involvement in research. Evaluation of the RAT will help us to understand the acceptability and appropriateness of the training, findings will be published to contribute to advancing the knowledge base of research training methods for involvement in general and for this particular population group in particular. The study will facilitate better understanding of best practice for the involvement of older people with dementia and age-related hearing and/or vision impairment in research.

## Additional files

Additional file 1: Support and Learning Needs Form. Support and Learning Needs Form used during introductory meeting with RUG members to establish individual needs/preferences for resources during RUG meeting and training sessions. (DOCX 69 kb)

Additional file 2: RUG Members Interview Topic guide. RUG one to one semi structured interview guide (DOCX 16 kb)

Additional file 3: RUG Members Focus Groups Interview guide. Focus group interview guide. (DOCX 12 kb)

Additional file 4: SENSE-Cog Researchers Interview Guide. One to one semi structured interview guide. (DOCX 12 kb)

Additional file 5: Easy access participant information sheet and consent form. Examples of the easy access participant information sheet and consent form used with SENSE-Cog RUGs to ensure informed participation in the evaluation study. (DOCX 3311 kb)

## Abbreviations

EQUIP: Enhancing the Quality of User Involved Care Planning
GRIPP: Guidance for Reporting Involvement of Patients and the Public
MICRA: Manchester Institute for Collaborative Research on Ageing
RUG: Research User Group
TARS: Training Acceptability Rating Scale

## References

[1] Alzheimers’ Research UK. https://www.dementiastatistics.org/statistics/global-prevalence (2016) Accessed 28th March 2018.

[2] Gove D, Diaz-Ponce A, Georges J, Moniz-Cook E, Mountain G, Chattat R (2018). Øksnebjerg L; European working Group of People with dementia. Alzheimer Europe's position on involving people with dementia in research through PPI (patient and public involvement). Aging Mental Health.

[3] Meiland Franka, Innes Anthea, Mountain Gail, Robinson Louise, van der Roest Henriëtte, García-Casal J Antonio, Gove Dianne, Thyrian Jochen René, Evans Shirley, Dröes Rose-Marie, Kelly Fiona, Kurz Alexander, Casey Dympna, Szcześniak Dorota, Dening Tom, Craven Michael P, Span Marijke, Felzmann Heike, Tsolaki Magda, Franco-Martin Manuel (2017). Technologies to Support Community-Dwelling Persons With Dementia: A Position Paper on Issues Regarding Development, Usability, Effectiveness and Cost-Effectiveness, Deployment, and Ethics. JMIR Rehabilitation and Assistive Technologies.

[4] Dewing J (2002). From ritual to relationship: a person-centred approach to consent in qualitative research with older people who have a dementia. Dementia.

[5] Hubbard G, Downs M, Tester S (2003). Including older people with dementia in research: challenges and strategies. Aging Ment Health.

[6] Murphy K, Jordan F, Hunter A, Cooney A, Casey (2015). Articulating the strategies for maximising the inclusion of people with dementia in qualitative research studies. Dementia (London).

[7] Morgan N, Grinbergs-Saull A, Murray M. We can make our research meaningful’. The impact of the Alzheimer’s Society Research. 2018. http://slginvolvement.org.uk/wp-content/uploads/2018/05/Research-Network-Report-low-res-public.pdf. Accessed 23 Sept 2018.

[8] INVOLVE. Briefing notes for researchers. INVOLVE. Eastleigh. UK. http://www.invo.org.uk/wp-content/uploads/2012/04/INVOLVEBriefingNotesApr2012.pdf (2012) Accessed 4 Mar 2018

[9] Going the Extra Mile a strategic review of public involvement in the National Institute for Health Research. https://www.nihr.ac.uk/patients-and-public/documents/Going-the-Extra-Mile.pdf (2015) Accessed 4 Mar 2018.

[10] Alzheimer's Society. Outcomes of the James Lind Alliance. Dementia priority setting partnership. 2013. https://www.alzheimers.org.uk/download/downloads/id/2226/outcomes_from_the_james_lind_alliance_priority_setting_partnership.pdf Accessed 11 Mar 2018.

[11] Deane Katherine H O, Flaherty Helen, Daley David J, Pascoe Roland, Penhale Bridget, Clarke Carl E, Sackley Catherine, Storey Stacey (2014). Priority setting partnership to identify the top 10 research priorities for the management of Parkinson's disease. BMJ Open.

[12] Mockford C, Murray M, Seers K, Oyebode J, Grant R, Boex S, Staniszewska S, Diment Y, Leach J, Sharma U, Clarke R, Suleman R. A SHARED study: the benefits and costs of setting up a health research study involving lay co-researchers and how we overcame the challenges. Research Involvement and Engagement. 2016;2(8): 10.1186/s40900-016-0021-3. 10.1186/s40900-016-0021-3 PMC5611649 29062509

[13] Poland F, Mapes S, Pinnock H, Katona C, Sorensen S, Fox C, Maidment ID (2014). Perspectives of carers on medication management in dementia: lessons from collaboratively developing a research proposal. BMC Res Notes.

[14] Paterson Charlotte, A Allen Jeffrey, Browning Margaret, Barlow Gillian, Ewings Paul (2005). A pilot study of therapeutic massage for people with Parkinson's disease: the added value of user involvement. Complementary Therapies in Clinical Practice.

[15] Yates LA, Orrell M, Spector A, Orgeta V (2015). Service users' involvement in the development of individual cognitive stimulation therapy (iCST) for dementia: a qualitative study. BMC Geriatr.

[16] Bunn F, Sworn K, Brayne C, Iliffe S, Robinson L, Goodman C (2015). Contextualizing the findings of a systematic review on patient and carer experiences of dementia diagnosis and treatment: a qualitative study. Health Expectations: An International Journal of Public Participation in Health Care & Health Policy.

[17] Martin S, Fleming J, Cullum S, Dening T, Rait G, Fox C, Katona C, Brayne C, Lafortune L. Exploring attitudes and preferences for dementia screening in Britain: contributions from carers and the general public. BMC Geriatrics. 2015;15(110) doi: 10.1186/s12877-015-0100-6 10.1186/s12877-015-0100-6 PMC4564978 26354754

[18] Brooks J, Savitch N, Gridley K. Removing the 'gag' : Involving people with dementia in research as advisers and participants. Social Research Practice. The SRA journal for methods in applied social research. 2017;3:3–14.

[19] Stevenson M, Taylor B. Involving individuals with dementia as co-researchers in analysis of findings from a qualitative study. Dementia. 2017:1–12 10.1177/1471301217690904. 10.1177/1471301217690904 28133983

[20] Littlechild Rosemary, Tanner Denise, Hall Kelly (2014). Co-research with older people: Perspectives on impact. Qualitative Social Work: Research and Practice.

[21] Hanson Elizabeth, Magnusson Lennart, Arvidsson Helene, Claesson Anette, Keady John, Nolan Mike (2007). Working together with persons with early stage dementia and their family members to design a user-friendly technology-based support service. Dementia.

[22] SENSE-Cog. https://www.sense-cog.eu/ (2016) Accessed 26 Mar 2018.

[23] Enhancing the Quality of User Involved Care Planning in Mental Health Services (EQUIP) (2012) http://research.bmh.manchester.ac.uk/equip. Accessed 2 Apr 2018

[24] Public Programmes Team. https://research.cmft.nhs.uk/facilities-services/public-programmes. (2018) Accessed 2 Apr 2018.

[25] Milne D, Noone S (1996). Teaching and training for non-teachers.

[26] Grundy A, Walker L, Meade O, Fraser C, Cree L, Bee P, Lovell K, Callaghan P (2017). Evaluation of a co-delivered training package for community mental health professionals on service user- and carer-involved care planning. J Psychiatry Mental Health Nurs.

[27] Carpenter J, Milne D, Lombardo C, Dickinson C (2007). Process and outcomes of training in psychosocial interventions in mental health: a stepwise approach to evaluation. J Ment Health.

[28] WHO. Process of translation and adaptation of instruments. 2018 http://www.who.int/substance_abuse/research_tools/translation/en/ Accessed 28^th^ September 2018.

[29] Guest Greg, Bunce Arwen, Johnson Laura (2006). How Many Interviews Are Enough?. Field Methods.

[30] O’Reilly Michelle, Parker Nicola (2012). ‘Unsatisfactory Saturation’: a critical exploration of the notion of saturated sample sizes in qualitative research. Qualitative Research.

[31] Using Excel for Qualitative Data Analysis. http://www.academia.edu/5062702/Using_Excel_for_Qualitative_Data_Analysis. (2018) Accessed 30 April 2018.

[32] Ritchie J, Lewis J (2003). Qualitative research practice: a guide for social science students and researchers.

[33] Braun V, Clarke V (2006). Using thematic analysis in psychology. Qual Res Psychol.

[34] Staniszewska S, et al. GRIPP2 reporting checklists: tools to improve reporting of patient and public involvement in research. BMJ. 2017;358. 10.1136/bmj.j3453. 10.1136/bmj.j3453 PMC5539518 28768629

[35] Mental capacity Act 2005: http://www.justice.gov.uk/downloads/protecting-the-vulnerable/mca/mca-code-practice-0509.pdf (2013) Accessed 5th March 2018.

[36] Miah J, Dawes P, Leroi I, Starling B, Edwards S, Parsons S (2018). Types and impact of patient and public involvement in dementia research and clinical service development in Europe: a systematic literature review.

[37] Brett J, Staniszewska S, Mockford C, Herron-Marx S, Hughes J, Tysall C, Suleman R (2014). Mapping the impact of patient and public involvement on health and social care research: a systematic review. Health Expect.

[38] Domecq JP, Prutsky G, Elariyah T, Wang Z, Nabhan M, Shippee N (2014). Patient engagement in research: a systematic review. Health Services Research BMC.

[39] Staley K. Exploring Impact: Public involvement in NHS, public health and social care research. Eastleigh: INVOLVE. http://www.invo.org.uk/posttypepublication/exploring-impact-public-involvement-in-nhs-public-health-and-social-care-research/. (2009) Accessed 28 Mar 2018.

[40] Telford R, Boote J, Cooper C (2004). What does it mean to involve consumers successfully in NHS research? A consensus study. Helath Expectations.

[41] Staniszewska S, Adebajo A, Barber R, Beresford P, Brady L-M, Brett J (2011). Developing the evidence base of patient and public involvement in health and social care research: the case for measuring impact. Int J Consum Stud.

[42] Littlechild Rosemary, Tanner Denise, Hall Kelly (2014). Co-research with older people: Perspectives on impact. Qualitative Social Work: Research and Practice.

[43] Tooke J (2013). Involving people with dementia in the work of an organisation: service user review panels. Quality in Ageing and People.

[44] Tanner D (2012). Co-research with older people with dementia: experience and reflections. J Mental Health.

[45] Cheston R, Bender M, Byatt S (2000). Involving people who have dementia in the evaluation of services: a review. J Ment Health.

[46] Acton GJ, Mayhew PA, Hopkins BA, Yauk S (1999). Communicating with individuals with dementia: the impaired person’s perspective. J Gerontol Nurs.

[47] McKeown J, Clarke A, Ingleton C, Repper J (2010). Actively involving people with dementia in qualitative research. Journal of Clincal Nursing.

[48] Clare L, Rowlands J, Bruce E, Surr C, Downs M. The Experience of Living with Dementia in Residential Care: An Interpretative Phenomenological Analysis. The Gerontologist:2008;48(6). 10.1093/geront/48.6.711 19139245

[49] Hellstrom I, Nolan M, Nordenfelt L, Lundh U (2007). Ethical and methodological issues in interviewing persons with dementia. Nurs Ethics.

[50] Downs M (1997). The emergence of the person in dementia research. Ageing Soc.

[51] Nygard L (2006). How can we get access to the experiences of people with dementia?. Scand J Occup Ther.

[52] Kitwood T (1997). Dementia reconsidered: the person comes first (rethinking ageing).

[53] Bartlett R (2012). Modifying the diary interview method to research the lives of people with dementia. Qualitative health research..

