# Supplementary material for: A protocol to evaluate the impact of involvement of older people with dementia and age-related hearing and/or vision impairment in a multi-site European research study
Source: Res Involv Engagem. 2018 Nov 22;4:44. doi: 10.1186/s40900-018-0128-9 (PMC6251148; doi:10.1186/s40900-018-0128-9)
Supplement: Supplementary file 1 — Support and Learning Needs Form. Support and Learning Needs Form used during introductory meeting with RUG members to establish individual needs/preferences for resources during RUG meeting and training sessions. (DOCX 69 kb) [file 40900_2018_128_MOESM1_ESM.docx]

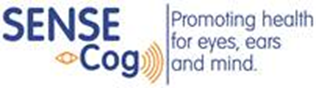
 **Research User Group (Site Name)**

**Needs and Support:**

1. **My Personal details:**

| Title and full name |  | |
| --- | --- | --- |
| Contact Details: what’s’ the best way to contact you? | Postal Address |  |
|  | Contact Number |  |
|  | Email |  |
|  | Other |  |

1. **My Needs and Support as a Research User Group member**

|  |
| --- |

1. **My Needs and Support for Learning for Research Awareness Training**

|  |
| --- |

1. **Other e.g. dietary requirements, travel arrangements**

|  |
| --- |
