# Supplementary material for: A protocol to evaluate the impact of involvement of older people with dementia and age-related hearing and/or vision impairment in a multi-site European research study
Source: Res Involv Engagem. 2018 Nov 22;4:44. doi: 10.1186/s40900-018-0128-9 (PMC6251148; doi:10.1186/s40900-018-0128-9)
Supplement: Supplementary file 2 — RUG Members Interview Topic guide. RUG one to one semi structured interview guide (DOCX 16 kb) [file 40900_2018_128_MOESM2_ESM.docx]

RUG Members Interview Topic guide

*Questions to be paraphrased by patient and public involvement coordinators

1. What did you think about the Research Awareness Training?

2. Do you feel the Research Awareness Training sessions helped you in your role as a member of the Research User Group?

3. Did you feel that your thoughts / input were listened to and valued?

4. Did you feel that your thoughts / input where useful to the SENSE-Cog research?

5. Were you given feedback from SENSE-Cog researchers / coordinators on where the Research User Group member’s had had an impact?

6. Do you feel your experience of being a Research User Group member matched up to how the role was originally described to you? Please explain:

7. To what extent do you feel you were able to contribute to the involvement tasks relating to the SENSE-Cog programme?

8. To what extent do you feel your involvement:

- Impacted on the different tasks within SENSE-Cog?
- Will impact on for the end users (older people with dementia and age-related hearing and/or vision impairment)?

9. In terms of your role as a Research User Group member within SENSE-Cog, to what extent do you feel you were:

a) Valued as a partner in this process?

b) Supported to get involved in the different tasks and opportunities within SENSE-Cog?

10. Thinking about your involvement in the different tasks can you talk a bit about your relationship with:

a) The researchers, how they supported you and communicated with you?

b) The Research User Group coordinators, how they supported you and communicated with you?
