# Supplementary material for: A protocol to evaluate the impact of involvement of older people with dementia and age-related hearing and/or vision impairment in a multi-site European research study
Source: Res Involv Engagem. 2018 Nov 22;4:44. doi: 10.1186/s40900-018-0128-9 (PMC6251148; doi:10.1186/s40900-018-0128-9)
Supplement: Supplementary file 3 — RUG Members Focus Groups Interview guide. Focus group interview guide. (DOCX 12 kb) [file 40900_2018_128_MOESM3_ESM.docx]

RUG Members Focus group Interview Topic guide

*Questions to be paraphrased by patient and public involvement coordinators

1. Tell us about your experience of the Research Awareness Training to date?

• What do you think of it?

• Do you feel the Research Awareness Training is helping you in your role as a member of the Research User Group?

• Is there anything that we should do differently?

• What aspects of the training did you like the most?

2. Do you feel your experience of being a Research User Group member matched up to how the role was originally described to you?

3. To what extent do you feel you were able to contribute to the involvement tasks relating to the SENSE-Cog programme?

• Did you feel that your thoughts / input where listened to and valued?

• Did you feel that your thoughts / input where useful to the SENSE Cog research?

4. How do you think your involvement is impacting on SENSE-Cog?

5. Were you given feedback from SENSE-Cog researchers / coordinators on where the Research User Group member’s had had an impact?

6. In terms of your role as a Research User Group member within SENSE-Cog, to what extent do you feel you were:

• Valued as a partner in this process?

• Supported to get involved in the different tasks and opportunities within SENSE-Cog?

• Empowered to get involved?

7. Thinking about your involvement in the different tasks can you talk a bit about your relationship with:

• The researchers, how they supported you and communicated with you?

• The Research User Group coordinators, how they supported you and communicated with you?

8. What are your thoughts on the venues used for the trainings and meetings?

9. Any other comments:
