# Supplementary material for: A protocol to evaluate the impact of involvement of older people with dementia and age-related hearing and/or vision impairment in a multi-site European research study
Source: Res Involv Engagem. 2018 Nov 22;4:44. doi: 10.1186/s40900-018-0128-9 (PMC6251148; doi:10.1186/s40900-018-0128-9)
Supplement: Supplementary file 4 — SENSE-Cog Researchers Interview Guide. One to one semi structured interview guide. (DOCX 12 kb) [file 40900_2018_128_MOESM4_ESM.docx]

SENSE-Cog Researchers Interview Guide

1. What is your role within the SENSE-Cog Programme?

2. Which work package/s are you involved in?

3. What are your views on involving Research User Groups?

4. Have you been involved in any previous work involving Research User Groups?

5. What has been you experience of involving Research User Groups in the SENSE-Cog research programme:

a) When were they involved?

b) What were they involved in? and how did this help?

c) Did you feel supported in involving Research User Groups?

d) Did you feel confident in involving Research User Groups?

6. In what ways do you think the involvement of Research User Groups has impacted on your work in the SENSE-Cog programme?

7. Do you think you will be involving Research User Groups in other areas of your future work programmes?

8. Any other comments?
