# Supplementary material for: A protocol to evaluate the impact of involvement of older people with dementia and age-related hearing and/or vision impairment in a multi-site European research study
Source: Res Involv Engagem. 2018 Nov 22;4:44. doi: 10.1186/s40900-018-0128-9 (PMC6251148; doi:10.1186/s40900-018-0128-9)
Supplement: Supplementary file 5 — Easy access participant information sheet and consent form. Examples of the easy access participant information sheet and consent form used with SENSE-Cog RUGs to ensure informed participation in the evaluation study. (DOCX 3311 kb) [file 40900_2018_128_MOESM5_ESM.docx]

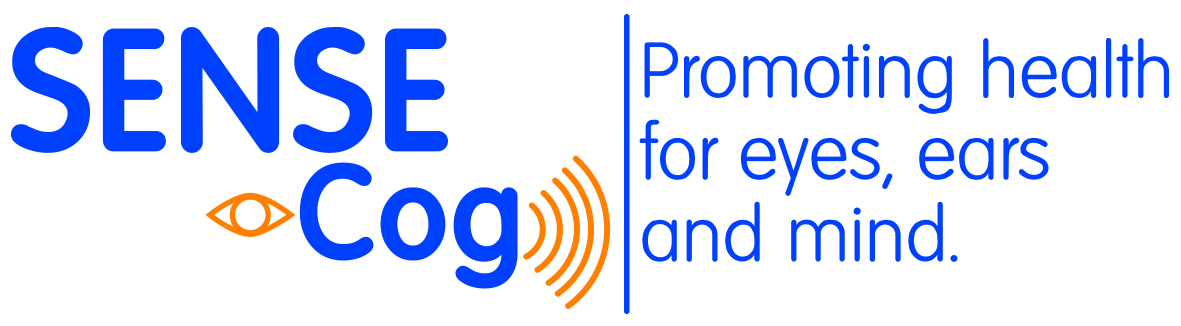
EASY-ACCESS PARTICIPANT INFORMATION SHEET

Research User Group participants impact in a multi-centre European dementia research programme

Steven Edwards,

Public Programmes Team,

Email: [steven.edwards@cmft.nhs.uk](mailto:steven.edwards@cmft.nhs.uk)

Telephone: 0161 276 3368

Jahanara Miah

Study Corodiantor

Unviersity of Manchester

Email: Jahanara.Miah@manchester.ac.uk Telephone: 0161 306 7911

1. Study Title: Research User Group participants impact in a multi-centre European dementia research programme


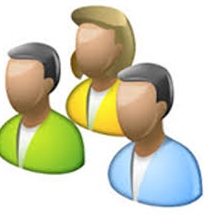

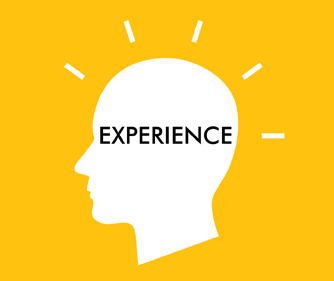


You are being invited to take part in a research study that aims to understand your experience as a Research User Group member and how you feel you have been able to contribute to the SENSE – Cog research programme.

2. You are invited to take part in the research study before you decide, it is important for you to understand why the research is being done and what it will involve.

Please take time to read the following information


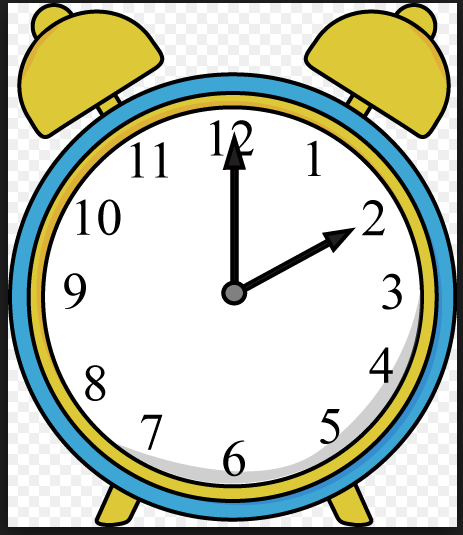

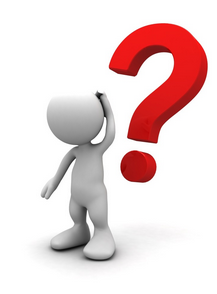


Please ask if there is anything that is not clear or if you would like more information.

3. What is the purpose of the research?

We want to understand Research User Group members’ experience of the SENSE-Cog research programme.


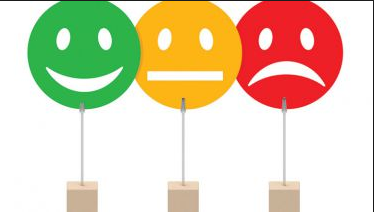


The findings from this study will help us understand how the involvement of Research User Groups has influenced our work within SENSE-Cog


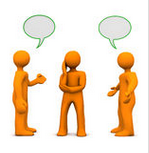

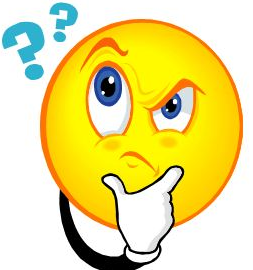


and also allow us to understand if the Research Awareness Training was helpful for the Research User Group members.

4. Why have I been chosen?

You are a SENSE-Cog Research User Group member


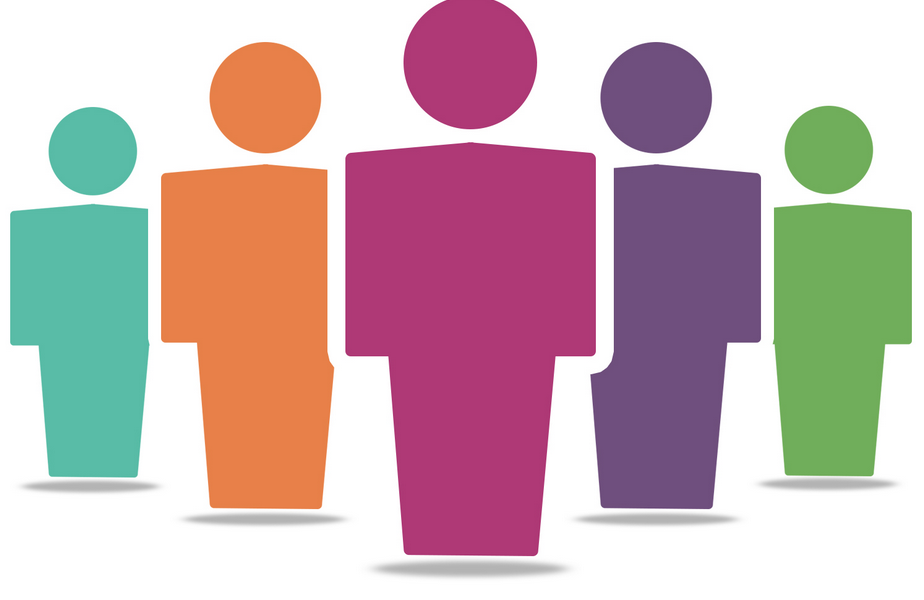


You have completed the Research Awareness Training


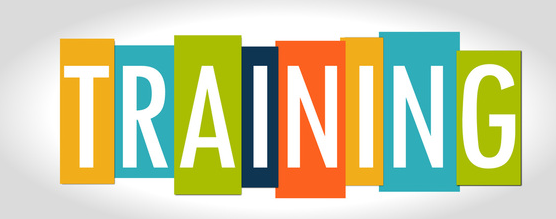


5. Do I have to take part?

No. It is up to you to decide whether or not to take part.

If you do decide to take part you will be given this information sheet to keep and be asked to sign a consent form.


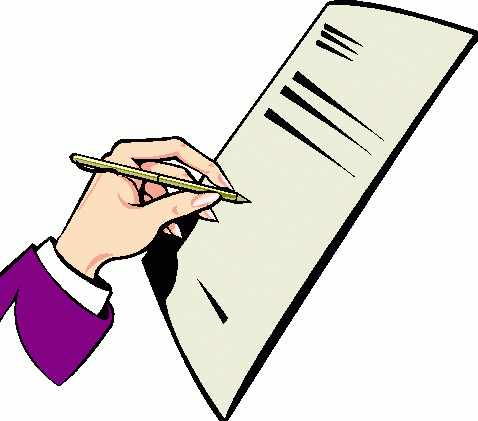


If you decide to take part you are still free to withdraw up to the point of anonymisation or after data analysis is complete without giving a reason and without detriment to yourself.

**6. What would I be asked to do if I took part?**

You will be asked to take part in a 60 minutes face-to-face interview with a researcher at University of Manchester.


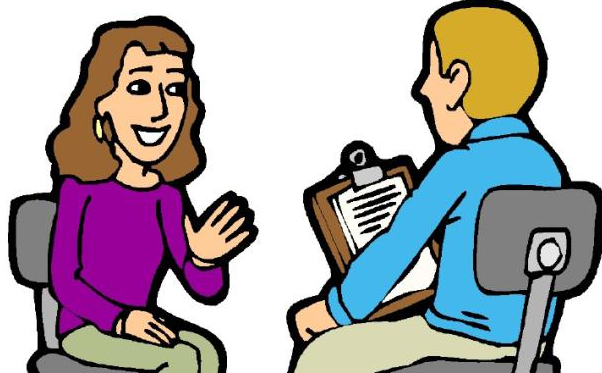


7. What happens to the data collected?

We will present the findings at conferences and publish them in scientific journals.


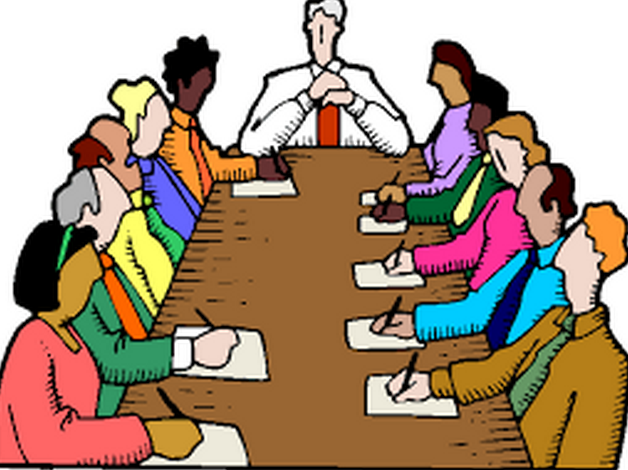

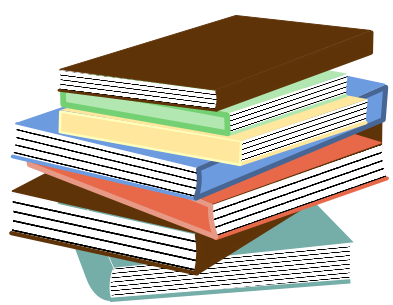


The data will be held securely for up to 10 years at the University of Manchester and will be destroyed as soon as it is no longer needed.


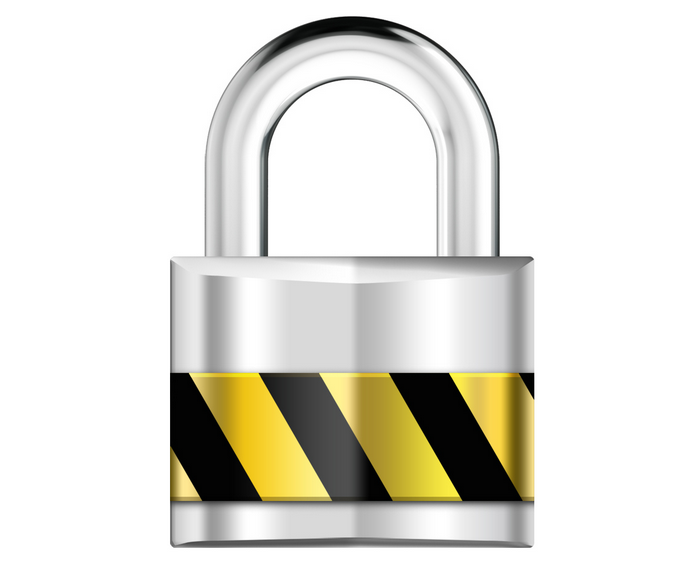

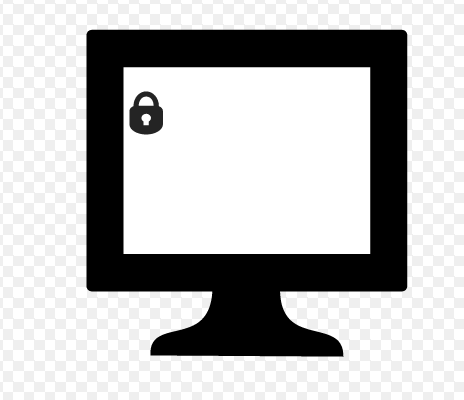


Individuals from the University or regulatory authorities may need to access the information collected to make sure we have conducted the research properly.

**8. How is confidentiality maintained?**

Your participation and all the information we collect about you will be kept confidential. Personal information will be kept securely and destroyed when not needed.
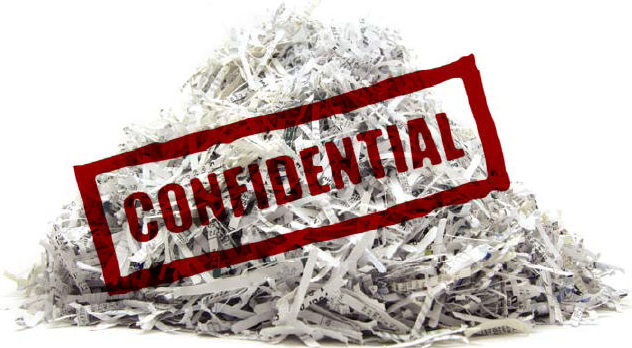


Data gathered from the interview will be anonymous. Only authorised people will be allowed to see your information.


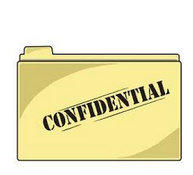

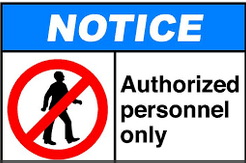


**9. What happens if I do not want to take part or if I change my mind?**

If you decide to take part you are still free to withdraw up to the point of anonymisation or after data analysis is complete without giving a reason and without detriment to yourself.


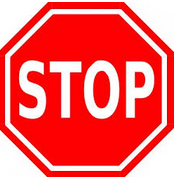

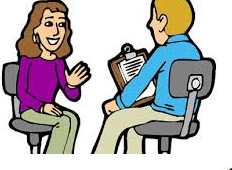

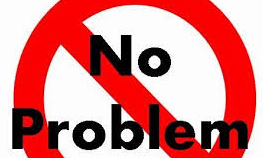


**10. Will I be paid for participating in the research?**

You will be compensated for your time and inconvenience, and we will arrange your travel arrangements.


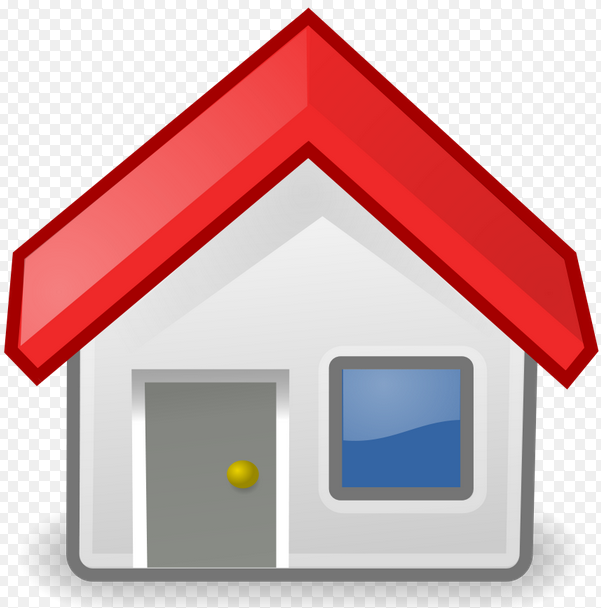

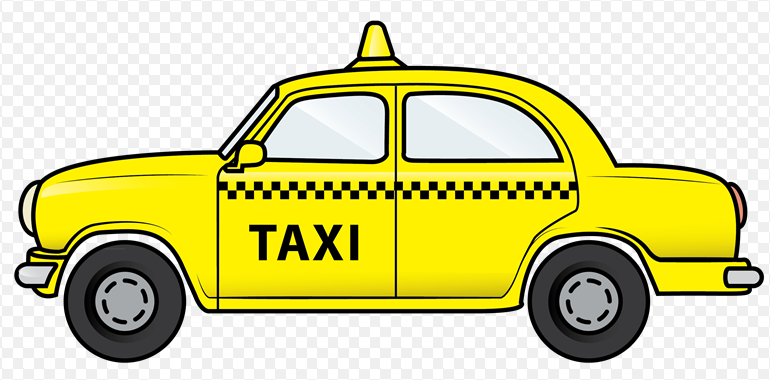


**8. What are the risks of taking part?**

We do not anticipate there to be any risks to participation. You may pause or stop the assessments at any time.


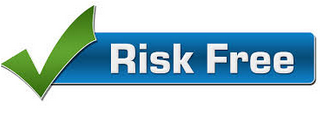


**9. What are the benefits of taking part?**

Taking part may not directly benefit you but will help us understand the role of Research User Groups within our research.

**10. Who has reviewed the research project**?

This research has been looked at by a Research Ethics Committee to protect your safety, rights, well-being and dignity.


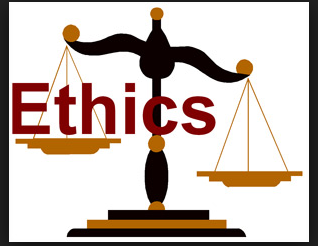


**11. What if something goes wrong?**

In the event that something does go wrong and you are harmed during the research you may have grounds for a legal action for compensation against the University of Manchester but you may have to pay your legal costs.
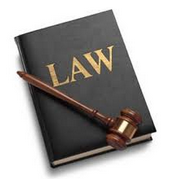


**12. What if I need more information or there is a problem?**

If you need more information or you’re not happy about the research
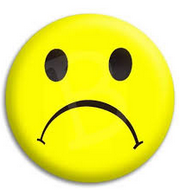


Please contact:
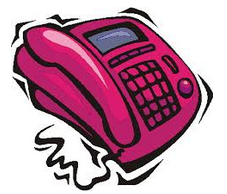
 **Jahanara Miah** by emailing: **Jahanara.Miah@manchester.ac.uk** or by telephoning **0161 306 7911**

**13. What Do I Do Now?**

If you decide you would like to take part, please read and sign the consent form.


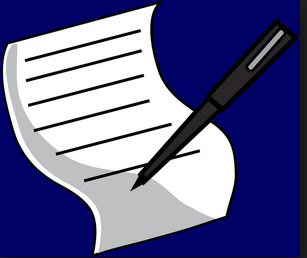


You will be given a copy of this information and signed consent form to keep.

Please take time to decide whether you want to take part.

Thank you for reading about this research.
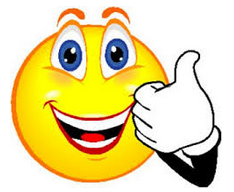


**This Project Has Been Approved by the University of Manchester’s Research Ethics Committee [UREC: 2017-0627-2142]**

**
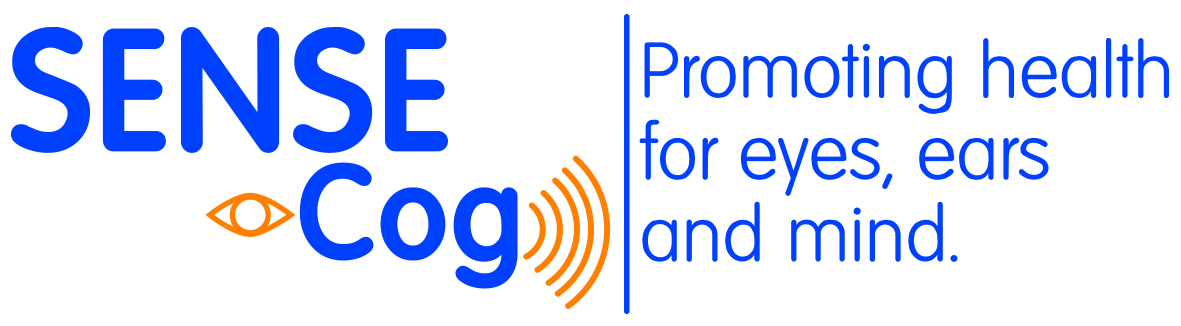
**

**Research User Group participants impact in a multi-centre European dementia research programme**

**Participant Information Sheet: RUG Focus Group Interview: version 1 : 28/07/17**

**You are being invited to take part in a research study that aims to understand your experience as a Research User Group member and how you feel you have been able to contribute to the SENSE – Cog research programme.**

**Before you decide, it is important for you to understand why the research is being done and what it will involve. Please take time to read the following information carefully and discuss it with others if you wish. Please ask if there is anything that is not clear or if you would like more information. Take time to decide whether or not you wish to take part. Thank you for taking the time to read this.**

**Who will conduct the research?**

**Steven Edwards, Public Programmes Team, The Nowgen Centre, 29 Grafton Street, Manchester, M13 9WU, Telephone 0161 276 3368**

**What is the purpose of the research?**

**This study aims to understand Research User Group members’ experience of the SENSE-Cog research programme. The findings from this study will help us understand how the involvement of Research User Groups has influenced our work within SENSE-Cog and also allow us to understand if the Research Awareness Training was helpful for the Research User Group members.**

**Why have I been chosen?**

**You have been invited because you have been a member of a SENSE-Cog Research User Group and you have completed Research Awareness Training sessions.**

**What would I be asked to do if I took part?**

**You will be asked to take part in a 1-hour long face-to-face interview with a researcher at University of Manchester.**

**What happens to the data collected?**

**We will present the findings at conferences and publish them in scientific journals. The data will be held securely for up to 10 years at the University of Manchester and will be destroyed as soon as it is no longer needed. Individuals from the University or regulatory authorities may need to access the information collected to make sure we have conducted the research properly.**

**How will the study benefit me?**

**Taking part in this study may not directly benefit you however your involvement will help us to understand if the Research Awareness Training is effective in providing the skills and knowledge to enable Research User Group members take a meaningful and active role in research. We do not anticipate there to be any risks to participation. You may pause or stop the interview at any time.**

**How is confidentiality maintained?**

**The data collected for this study will be stored securely and only the researchers conducting this study will have access to this data:**

**o Audio recordings will be destroyed and/or deleted at the end of data analysis.**

**o Hard copies of questionnaires will be kept in a locked cabinet.**

**o The files on the computer will be encrypted (that is no-one other than the researcher will be able to access them) and the computer itself password protected.**

**o At the end of the study, hard copies of questionnaires will be kept securely in a locked cabinet for ten years. At the end of this period, they will be destroyed.**

**o The typed version of your interview will be made anonymous by removing any identifying information including your name. Anonymised direct quotations from your interview may be used in the reports or publications from the study, so your name will not be attached to them.**

**o All your personal data will be confidential and will be kept separately from your interview responses.**

**There are some limits to confidentiality: if what is said in the interview makes me think that you, or someone else, is at significant risk of harm, I will have to break confidentiality and speak to a member of staff about this. If possible, I will tell you if I have to do this**

**Individuals from the University or regulatory authorities may need to access the data collected to ensure that the study is being carried out properly. All individuals will be authorised representatives from each organisation and will have a duty of confidentiality to all research participants.**

**What happens if I do not want to take part or if I change my mind?**

**It is up to you to decide whether or not to take part. If you do decide to take part you will be given this information sheet to keep and be asked to sign a consent form. If you decide to take part you are still free to withdraw up to the point of anonymisation or after data analysis is complete without giving a reason and without detriment to yourself.**

**Will I be paid for participating in the research?**

**You will be compensated for your time and inconvenience, and we will arrange your travel arrangements.**

**What is the duration of the research?**

**You will be asked to attend one 60 minutes face to face interview with a researcher.**

**Where will the research be conducted?**

**The research will be conducted at Jean McFarlane Building, Oxford Road, University of Manchester, M13 9PL.**

**Will the outcomes of the research be published?**

**We will present the findings at conferences and publish them in scientific journals. We will also write to you (if you choose) and let you personally know the outcome of the study. If you would like this information or would like more information about future studies, please leave us your name and address. All your information will be anonymised and you will not be identified personally.**

**Who has reviewed the research project?**

**This research has been looked at by an independent group of people, called a Research Ethics Committee to protect your safety, rights, well-being and dignity. This study has been reviewed by the Committee [UREC: 2017-0627-2142].**

**What if something goes wrong?**

**In the event that something does go wrong and you are harmed during the research you may have grounds for a legal action for compensation against the University of Manchester but you may have to pay your legal costs.**

**What if I want to make a complaint?**

**If you have a concern about any part of this study, you should ask to speak to Jahanara Miah (Study Coordinator), Division of Neuroscience and Experimental Psychology, School of Biological Sciences, Room 3.309, Jean McFarlane Building, University of Manchester, Oxford Road, M13 9PL, by emailing: Jahanara.Miah@manchester.ac.uk or by telephoning 0161 306 7911**

**If you wish to make a formal complaint or if you are not satisfied with the response you have gained from the researchers in the first instance then please contact the Research Governance and Integrity Manager, Research Office, Christie Building, University of Manchester, Oxford Road, Manchester, M13 9PL, by emailing:** [**research.complaints@manchester.ac.uk**](mailto:research.complaints@manchester.ac.uk)**or by telephoning 0161 275 2674 or 275 2046.**

**What Do I Do Now?**

**If you have any queries about the study or if you are interested in taking part then please contact Steven Edwards, Public Programmes Team, The Nowgen Centre, 29 Grafton Street, Manchester, M13 9WU, by emailing** [**steven.edwards@cmft.nhs.uk**](mailto:steven.edwards@cmft.nhs.uk) **or by telephoning 0161 276 3368**

**This Project Has Been Approved by the University of Manchester’s Research Ethics Committee**

**[UREC: 2017-0627-2142]**

**
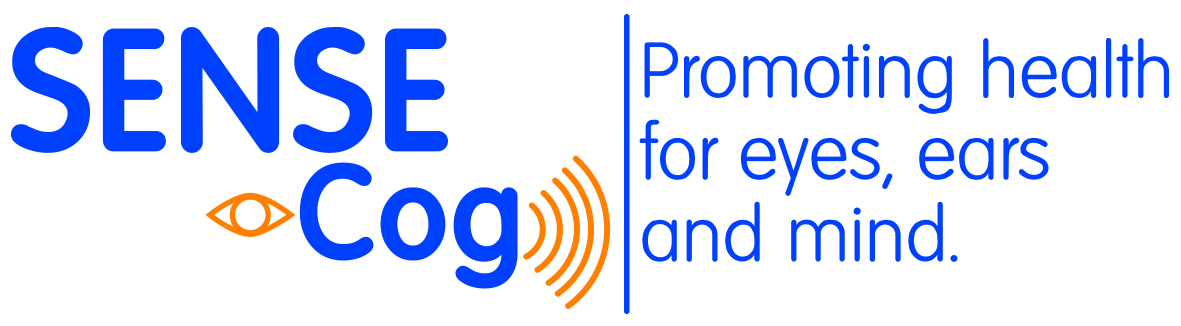
**

**Research User Group participants impact in a multi-centre European dementia research programme**

**CONSENT FORM : RUG Interview : version 2 : 08/03/17**

**If you are happy to participate please complete and sign the consent form below.**

**Please initial box**

| 1. **I confirm that I have read the attached information sheet on the above project and have had the opportunity to consider the information and ask questions and had these answered satisfactorily.** |  |
| --- | --- |
| 1. **I understand that my participation in the study is voluntary and that I am free to withdraw at any time up to the point of anonymisation or after data analysis is complete without giving a reason and without detriment to myself.** |  |
| 1. **I understand that the interviews will be audio-recorded.** |  |
| 1. **I understand that my data will remain confidential and anoymised.**     1. **I understand there are some limits to confidentiality: and if the interviewer thinks that I, or someone else, is at significant risk of harm, confidentiality maybe breached.** |  |
| 1. **I agree to the use of anonymous quotes.** |  |
| 1. **I understand that authorised individuals may require access to my data in order to monitor study conduct.** |  |

**I agree to take part in the above project**

|  |  |  |  |  |
| --- | --- | --- | --- | --- |
| **Name of participant** |  | **Date** |  | **Signature** |
| **Name of researcher** |  | **Date** |  | **Signature** |

**This Project Has Been Approved by the University of Manchester’s Research Ethics Committee**

**[UREC: 2017-0627-2142]**
